# Supplementary material for: Trabectedin Is Active against Two Novel, Patient-Derived Solitary Fibrous Pleural Tumor Cell Lines and Synergizes with Ponatinib
Source: Cancers (Basel). 2022 Nov 15;14(22):5602. doi: 10.3390/cancers14225602 (PMC9688590; doi:10.3390/cancers14225602)
Supplement: Supplementary file 1 [file cancers-14-05602-s001.zip › cancers-1936901-supplementary.pdf]

Supplementary Material

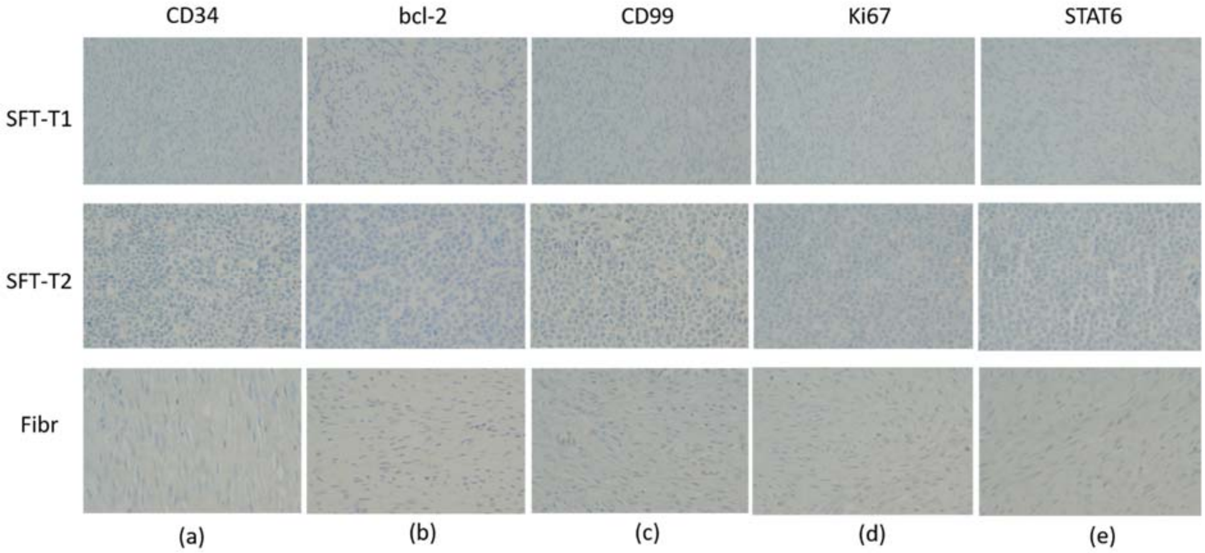

Figure S1. Representative (immune-) histological pictures of negative control stainings with isotypes corresponding to the antibodies (**a**) CD34, (**b**) bcl-2, (**c**) CD99, (**d**) Ki67, (**e**) STAT6 of the two included tumors (SFT-T1 and SFT-T2) and an excluded fibromatosis patient (Fibr) compare Figure 2 of the main text.

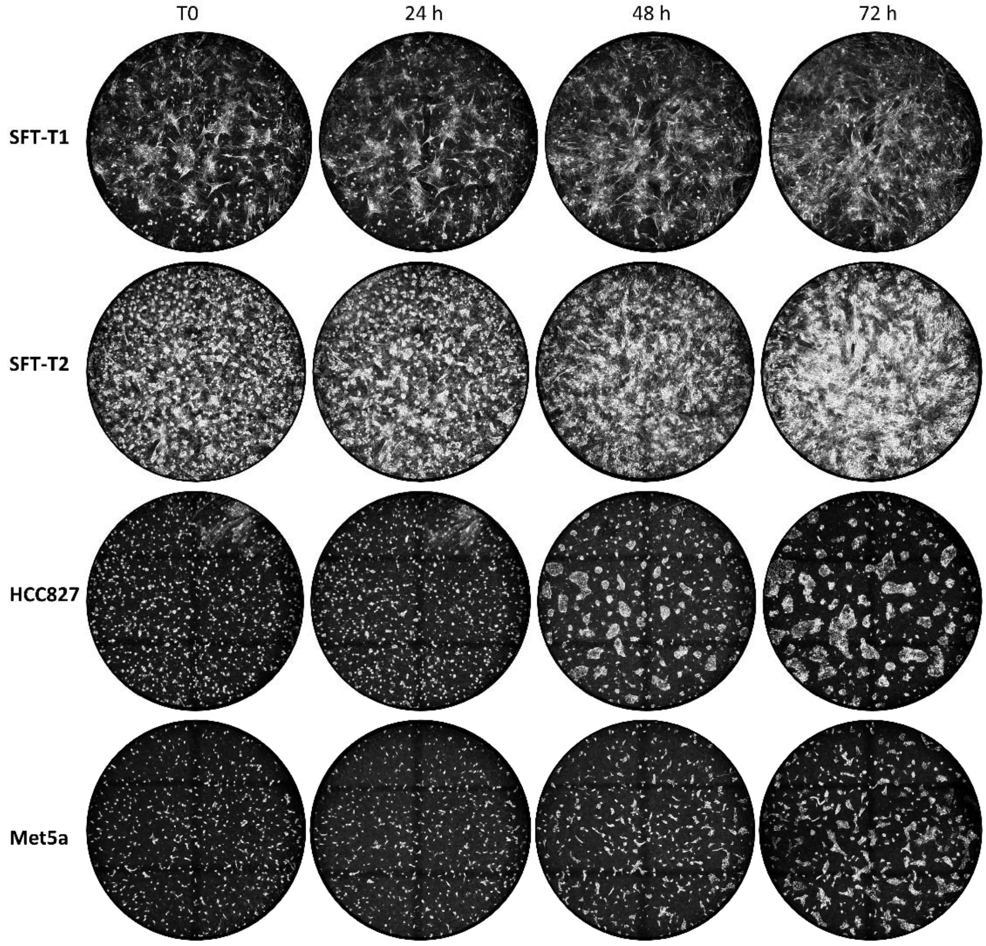

(a)

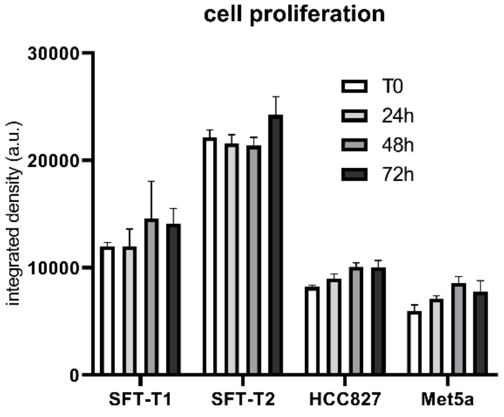

(b)

Figure S2. SFT-T1, SFT-T2, HCC827, and Met5a cells show stable *in vitro* growth. (**a**) Representative digital phase contrast images of respective cell lines after T0, 24, 48, and 72 h. (**b**) Quantification of pixel intensities of images shown in (a); n=3.

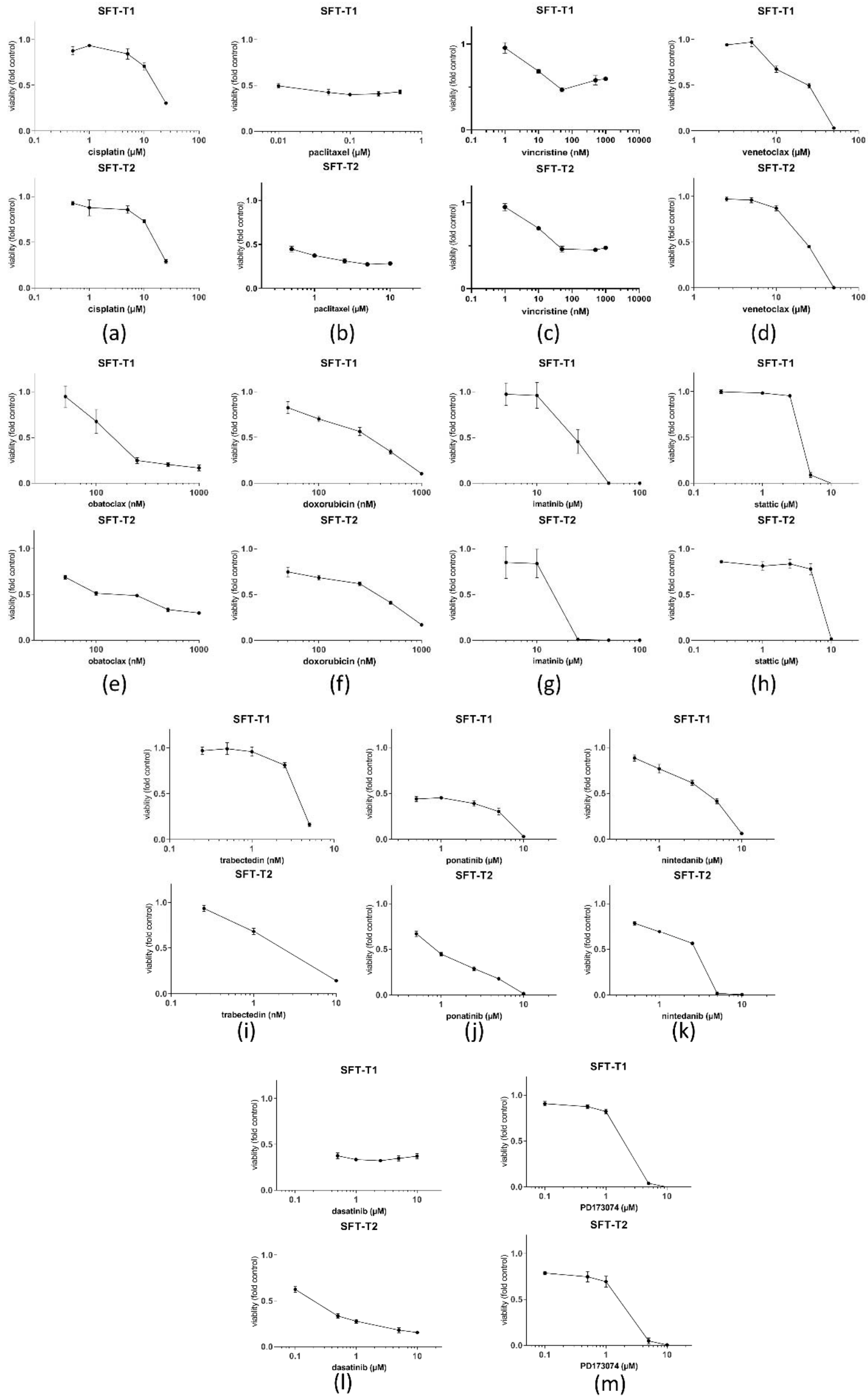

Figure S3. Representative *in vitro* dose-response curves of SFT-T1 (upper) and SFT-T2 (lower) cells after 72 h exposure to (**a**) cisplatin, (**b**) paclitaxel, (**c**) vincristine, (**d**) venetoclax, (**e**) obatoclax, (**f**) doxorubicin, (**g**) imatinib, (**h**) stattic, (**i**) trabectedin, (**j**) ponatinib, (**k**) nintedanib, (**l**) dasatinib, and (**m**) PD173074 determined by MTT assay.

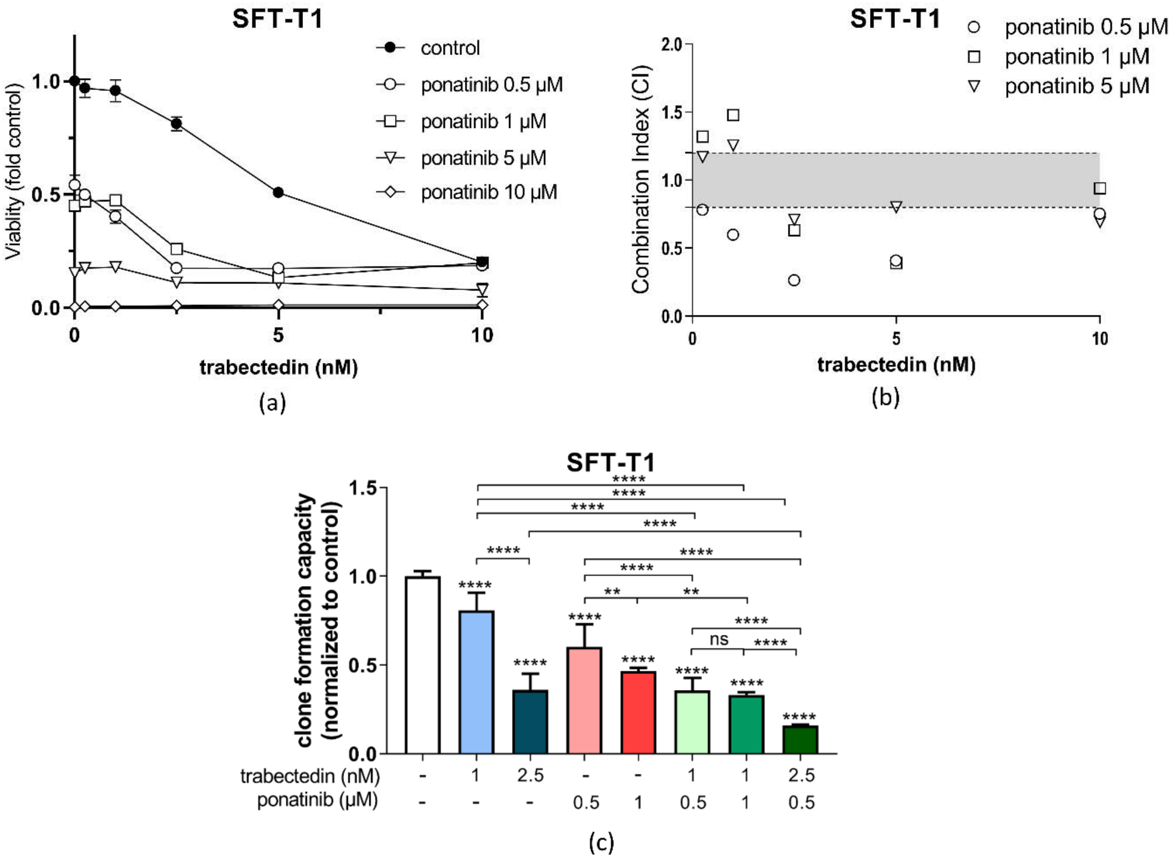

Figure S4. Ponatinib and trabectedin are active against low risk group SFT-T1 cell growth and synergize *in vitro*. (**a**) Combination of ponatinib with trabectedin shows synergistic anticancer activity. (**b**) Corresponding CI values. CI < 0.9, synergism; CI = 0.9-1.2, additive effects; or CI > 1.2 antagonism. (**c**) Quantification of effects of ponatinib and trabectedin as single agents and in combination on SFT-T1 clone formation capacity over a treatment period of nine days.

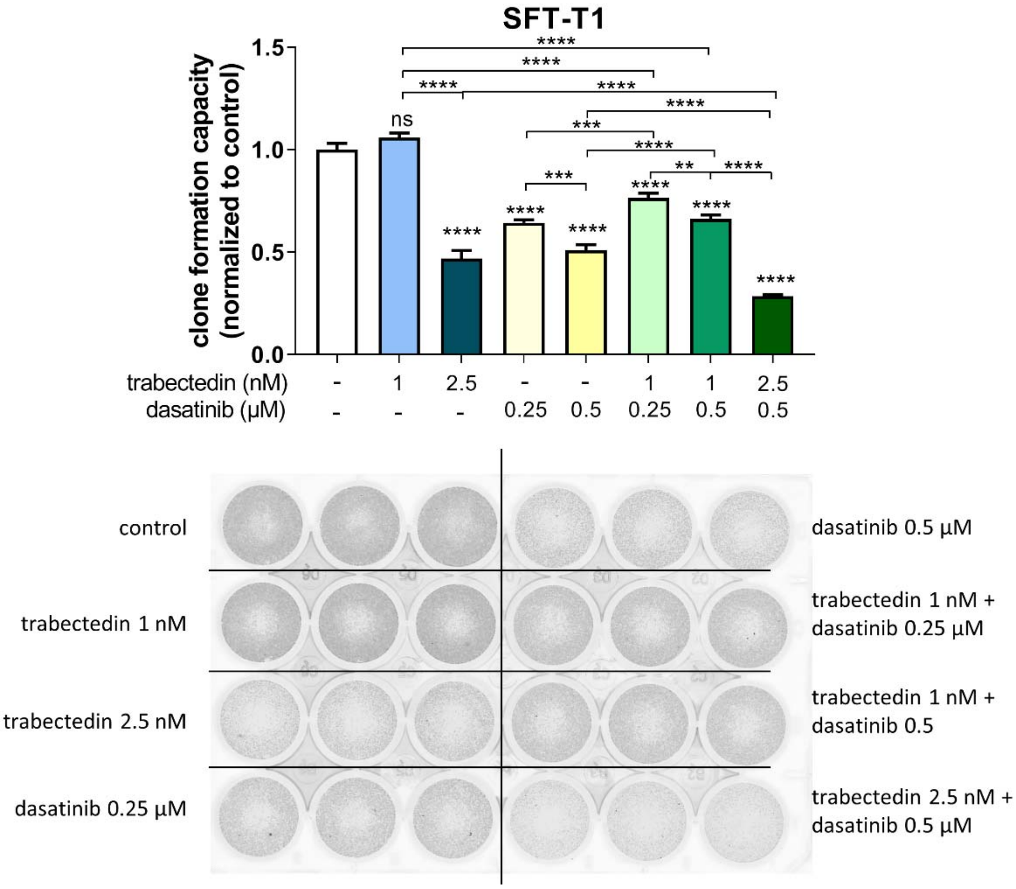

Figure S5. Dasatinib is active against SFT-T1 growth *in vitro* and enhances the anticancer activity of trabectedin. Effects of trabectedin as well as dasatinib as single agents and in combination on SFT clone formation capacity over a treatment period of nine days.

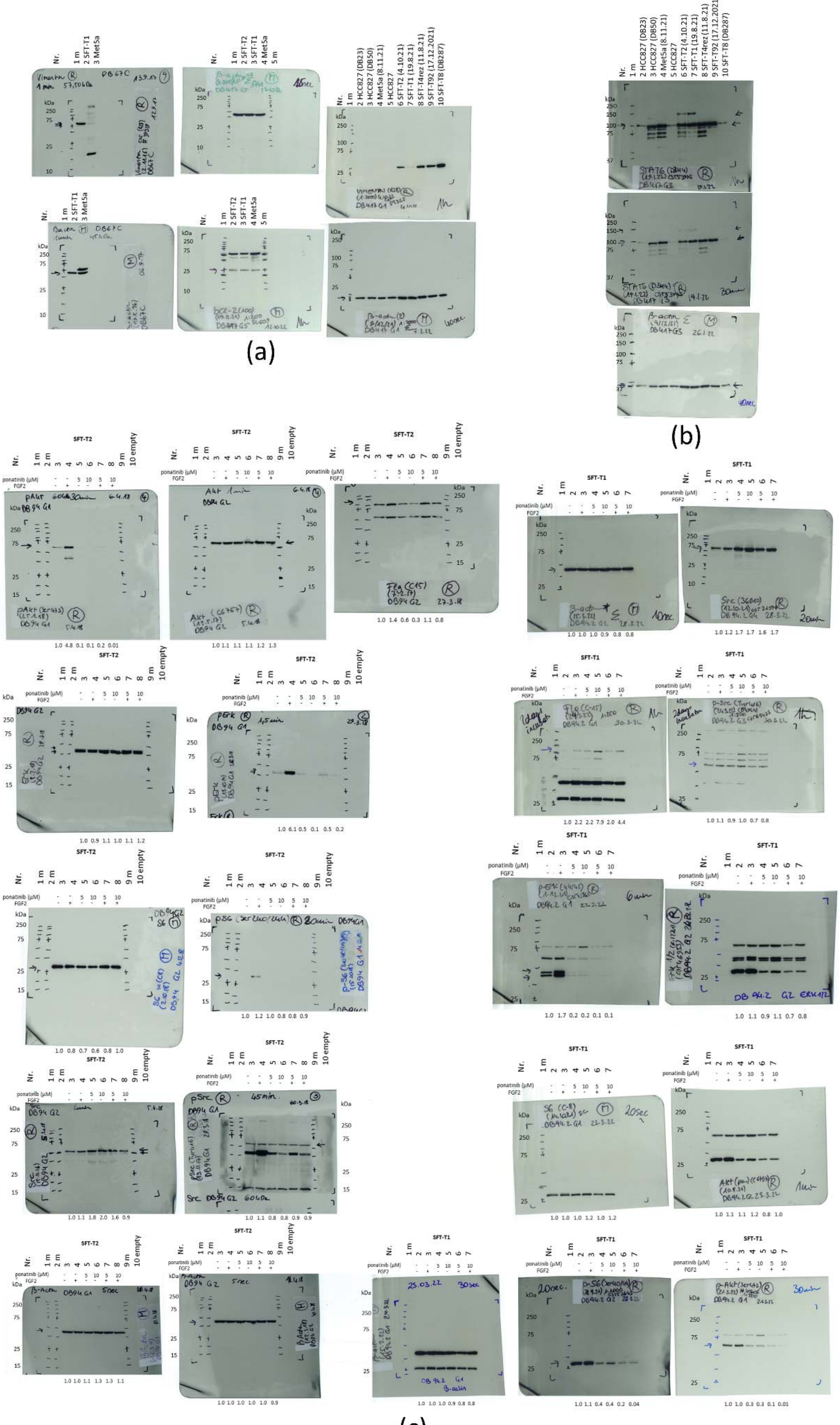
Nr.
1 m
2 SFT-T1
3 Met5a
1 m
2 SFT-T2
3 SFT-T1
4 Met5a
5 m
1 m
2 HCC827 (DB23)
3 HCC827 (DB50)
4 Met5a (8.11.21)
5 HCC827
6 SFT-T2 (4.10.21)
7 SFT-T1 (19.8.21)
8 SFT-T4rez (11.8.21)
9 SFT-T92 (17.12.2021)
10 SFT-T8 (DB287)
kDa
(a)
(b)
SFT-T2
SFT-T1
ponatinib (µM)
FGF2
10 empty
(c)

Figure S6. Original Western blots of (**a**) Figure 3b, (**b**) Figure 3d, and (**c**) Figure 6.
